# Prospective evaluation of the effect of early nasal layer closure on definitive repair in cleft palate patients

**DOI:** 10.1016/j.bjorl.2020.04.003

**Published:** 2020-05-05

**Authors:** Mohammed Abd-Alhussein Aljodah, Mustafa Zahi Al-Zajrawee

**Affiliations:** a University of Baghdad, Al-Kindy College of Medicine, Department of Surgery, Baghdad, Iraq; b University of Kufa, College of Medicine, Department of Surgery, Najaf, Iraq

**Keywords:** Cleft lip, Cleft palate, Nasal layer, Primary repair, Two-stage

## Abstract

**Introduction:**

The cleft palate is one of the most common congenital anomalies treated by plastic surgeons. The cleft width increases the tension of repair and necessitates excessive dissection that might affect maxillary growth. Decreasing the width of cleft minimize tension, dissection and may limit the impact on maxillary growth.

**Objectives:**

The purpose of the study was to evaluate the effect of nasal layer closure of the hard palate at the time of cleft lip repair in patients with complete cleft lip and palate, to demonstrate the efficacy of narrowing the gap and to reduce the incidence of fistulae or other complications.

**Methods:**

Thirty patients less than 1 year of age were included in this prospective observational study. A superiorly based vomer flap was used to repair the nasal layer of the cleft hard palate at the time of primary cleft lip repair. 12–14 weeks after the vomer flap, the cleft soft and hard palate was definitively repaired. Alveolar and palatal gaps were recorded during the 1st and 2nd operations to demonstrate the reduction of the gap defect.

**Results:**

The mean reduction of the alveolar cleft width in patients who had a vomer flap in the first stage was 4.067 mm and the mean reduction of the palatal gap was 4.517 mm. Only 3 patients developed small fistula on the repaired nasal layer that was discovered and corrected during definitive palatoplasty.

**Conclusion:**

Nasal layer closure is a simple surgical technique that can be used to close the hard palate at the time of cleft lip repair. It is a valuable addition to cleft lip and palate repair that may prevent some cleft palate surgical complications.

## Introduction

Cleft lip and palate are the most common congenital craniofacial anomalies treated by plastic surgeons.^1^ The incidence of cleft varies by race: it is estimated to be 1:750 live births in Caucasians, 1:2000 live births in patients of African descent, and 1:500 live births in those of Southeast Asian descent.^2^ Treatment of cleft palate has developed over an extended time. The goal in the design of the recent palatoplasty is no longer the perfect closure of the cleft palate but rather an optimal speech outcome without hazarding maxillofacial growth.^3^ The outcome of repairing the cleft defect depends on several factors such as cleft morphology, operator experience, selection of operative technique, and timing and sequence of surgical repair. The timing of treatment is the most variable factor, dependent upon parental preferences, including sometimes difficult discussions, and differing judgments that are not merely medical.^4^

The list of surgical techniques used in the palatal cleft is extensive. The repair differs depending upon whether the cleft is an isolated cleft palate or part of a unilateral or bilateral cleft lip and palate. The initial vomer flap, which was first introduced by Pichler in 1926, was defined as an inferiorly based pattern flap: an incision is created high on the septum, and the flap is reflected downward to give a single layer closure on the oral side.^3^, ^5^, ^6^ With this technique, some European centers noted a high percentage of maxillary retrusion, presumably from injury to the vomer-premaxillary suture, as well as a high fistula rate.^7^, ^8^ A similar problem has not been found with the superiorly based vomer flaps. This procedure includes reflecting the mucosa of the septum near the cleft margin, dissecting just sufficient to close the nasal mucosa of the opposite side. In the bilateral cleft palate, this requires a midline incision along the septum, and the two flaps are reflected in each direction. This procedure produces a two-layered closure with a low fistula rate and limited impact on maxillary growth.^9^

There are currently two common approaches to the timing of cleft palate repair: two-stage repair and single-stage repair.^5^ The dilemma of maxillary growth following cleft palate has directed some surgeons to support a two-stage repair. The general protocol, originally introduced by Schwekendiek and Doz, entailed repair of the soft palate at the same time as the cleft lip repair, around 4–6 months. The hard palate was obturated and repaired at about 4–5 years of age. Earlier ages have subsequently been proposed for hard palate repair, usually around 18–24 months. The rationale for this approach has been that the hard palate cleft narrows during the time between procedures, requiring less dissection and thus resulting in less maxillary growth disturbances.^10^

In this study, simultaneous repair of cleft lip and nasal layer of the hard palate by incorporating a superiorly based vomerine mucoperiosteal flap for patients who present with a complete cleft lip and palate has been adopted. The first stage was done at the time of cleft lip repair, and the second stage (which involves complete hard and soft palate repair) was done nearly 12–14 weeks after the first operation. This study aimed to evaluate the short-term effects of this repair, to show the efficacy of narrowing the gap and to reduce the incidence of fistulae or other complications.

## Patients and methods

### Study sample

This prospective study included 30 consecutive patients (18 females and 12 males) who presented with congenital complete unilateral cleft lip and palate who underwent surgical repair in the period between July 2017 and November 2019. A simultaneous vomer flap to repair the hard palatal defect was used at the time of lip repair. Inclusion criteria were the patients older than 2 months with complete unilateral cleft lip and palate had no other facial anomalies, nor any previous surgery or interventions. All patients were subjected to routine preparative examinations and investigations, including, hemoglobin level, bleeding profile, and virology screen.

### Ethical consideration

Ethical clearance for the study was obtained from the local institutional Scientific and Ethics Committee with approval number 32/2017 before the commencement of the study. All the patients’ parents have explained the study and operative procedures in detail with advantages and disadvantages, and they were included in the study only after giving informed written consent for publication of any accompanying photographs.

### Operative techniques and surgical steps

**Figure 1:**
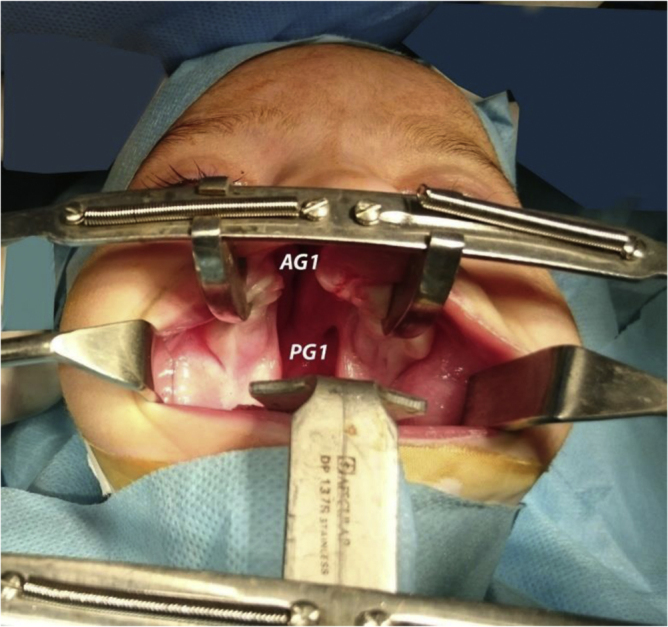
Measurement of alveolar gap (AG) and palatal gap (PG) at first surgery.

All operations were done under general anesthesia, with endotracheal intubation. Dingman's retractor was used to open the oral cavity and packing around the endotracheal tube was secured. To provide a quantitative argument to justify this study, Castroviejo Screw-Locking Caliper was used to measure the gap width pre-operatively in two points as shown in Fig. 1: the inter-alveolar gap (AG1), denoting the width of the gap between the alveolar ridge, and the inter-palatal gap (PG1), marking the gap between the posterior aspect of the hard palate.

**Figure 2:**
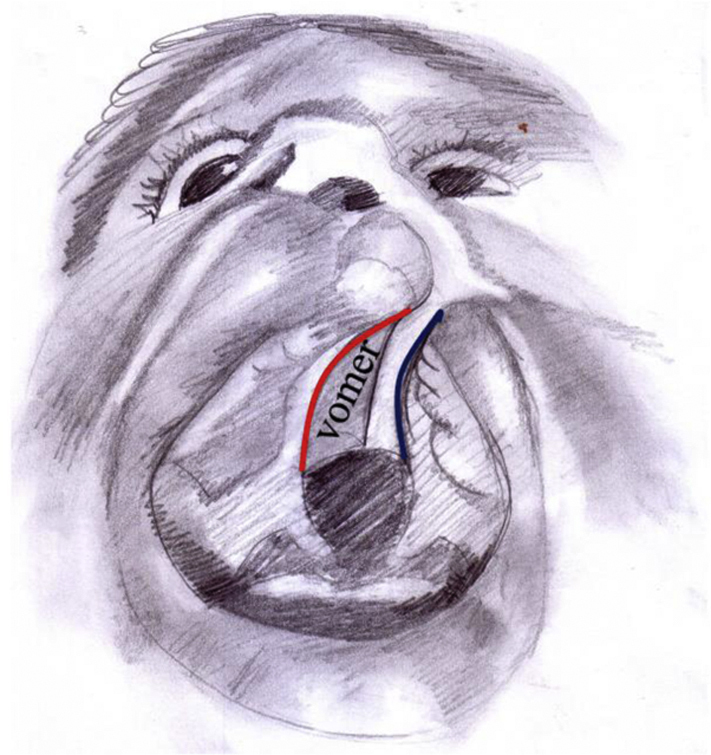
Scheme showing marking of dissecting vomer flap (red line), blue line is marking dissection of cleft side oral and nasal flaps for insetting the vomer flap.

We began by marking the anterior and posterior edges of the vomer, and the junction between the vomerine mucoperiosteal flap and the oral layer of the hard palate on the non-cleft side. Using methylene blue dye, we mark our incision starting at the alveolar cleft on the non-cleft side passing back through the junction between the vomer and the oral mucosa of the palatal shelf till the posterior end of the vomer (Fig. 2).

**Figure 3:**
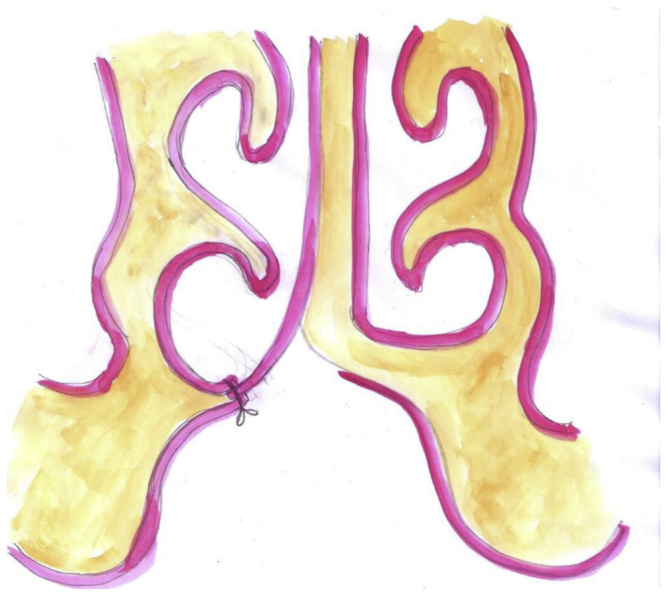
Elevation and pattern of suturing of vomer mucoperiosteal flap.

We added a back cut at the posterior edge of the vomer to ease the dissection, and to allow flipping the vomerine mucoperiosteal flap across the gap. After we finished the marking, infiltration with 1% lidocaine and 1/200,000 adrenaline was injected along the incision line and beneath the flap, to allow a bloodless dissection field, and to benefit from hydro-dissection. Using the n° 15 blade, we incised the vomer at the junction with palatal shelf, and the flap was then elevated cranially by using the periosteal elevator. We extended our dissection in a cranial direction just enough to allow flipping the flap horizontally across the cleft. Then the oral and nasal mucoperiosteal flap of the palatal shelf on the cleft side was dissected free using a periosteal elevator; usually dissection here is limited to no more than 5 mm just to allow insetting the vomerine flap sandwiched between these two layers (Fig. 3).

A 4-0 polyglycolic suture is used to stitch the vomerine flap to the dissected oral and nasal flap of the cleft side. The suture is fashioned in a horizontal mattress pattern, starting at the oral mucosa of the cleft through the vomerine flap, and going out through the nasal flap. Then going back the same pathway in reverse, going in from nasal layer tacking the vomer flap and out through the oral mucoperiosteal flap. This pattern of suture will allow the vomerine flap to insert sandwiched between the oral and nasal layer of the cleft side. Usually, three to four horizontal stitches are more than enough to secure the flap. Hemostasis is secured and the Dingman's retractor removed. Finally, the cleft lip repair was completed using the Modified Millard Rotation Advancement technique. Lip repair dressing was accomplished with steri-strips.

### Postoperative follow-up

**Figure 4:**
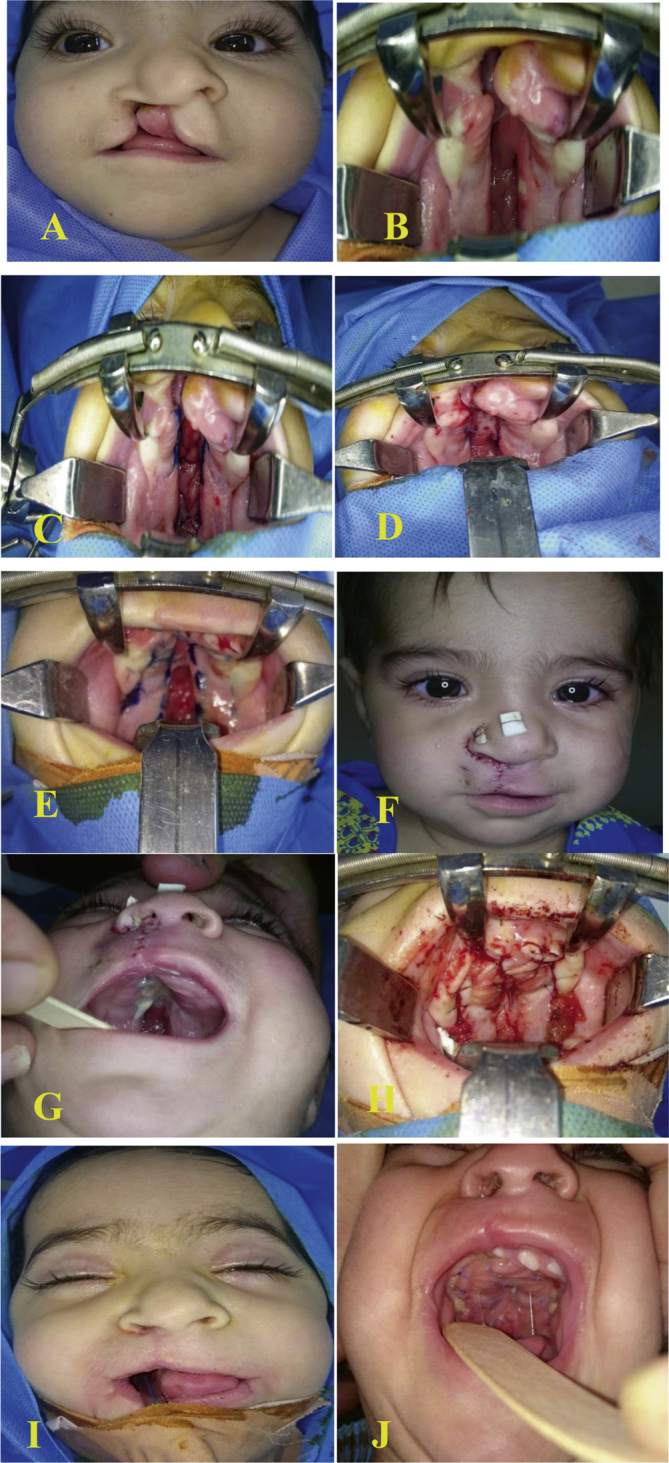
Six-month-old female presented with complete right cleft lip and palate (A) preoperatively, (B) through (D) vomer flap marking and insetting, (E) and (F) five days after surgery, (G) through (I) second stage complete palatal repair after 3 months, (J) five days after the second surgery.

Patients were kept on injectable antibiotics (3rd generation cephalosporins) for the first postoperative day and then discharged home on the second postoperative day on oral suspension antibiotic Cefixime 100 mg/5 mL. Parents were instructed to keep the child on a liquid diet, to feed him using a spoon followed by plain water for at least 3 weeks. On the 7th postoperative day, patients were seen for follow up, and for suture removal from the lip. The patient follow ups continue regularly every 2 weeks for the first month, then monthly until the time of definitive palatal repair, which was planned 12–14 weeks after the first surgery. During follow up, we checked for flap necrosis, bleeding, and the development of a fistula. In the second and the final surgery, the definitive palatal repair was accomplished by Bardach's two-flap technique and the soft palate was repaired in a straight line with a reconstruction of the muscle sling by intravelar veloplasty. The epithelialized area on the oral surface of vomer flap was carefully dissected to remove epithelium to allow contact of raw area between flaps. Measurement of the alveolar and palatal gaps (AG2 and PG2 respectively) was done to compare it with the first readings and to show the reduction in the gap width after the implication of vomer flap to the defect. The pre- and postoperative images of one patient from our cases are shown in Fig. 4.

### Data collection and analysis

In each case, information about the patient was obtained in the form of a preoperative questionnaire, included age, sex, address, mobile phone number, any family history of cleft, side of the cleft lip-palate, other associated congenital anomalies, other illness, physical findings, preoperative investigations, operative procedure, postoperative complications, and follow-up (12–14 weeks). All the information about each patient was obtained in a separate data sheet and arranged systemically and presented in tables. Statistical analyses were performed for intraoperative measurements. Continuous variables were expressed as mean ± SD. Paired *t*-test was used to analyze the continuous variables; *p*-value of less than 0.05 was considered to be statistically significant. Statistical analyses were performed with Statistical Package for Social Sciences (SPSS), SPSS® for Windows, version 19.0 (IBM Corp, Armonk, NY). Some preoperative, intraoperative and postoperative clinical photographs were also reclaimed and shown.

## Results

**Figure 5:**
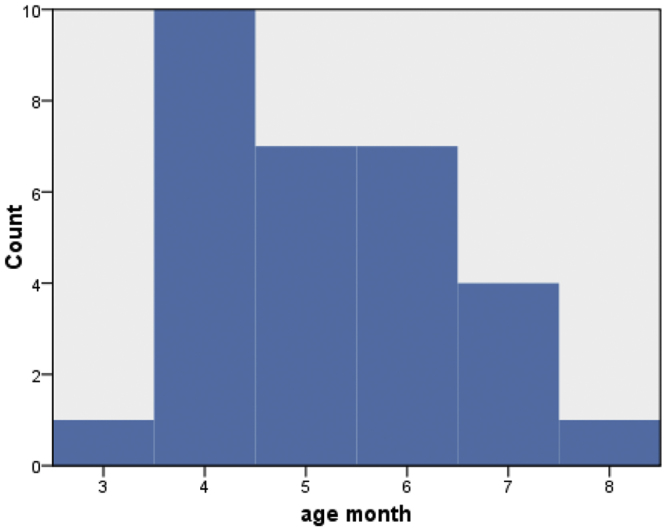
Age distribution of the study sample.

**Figure 6:**
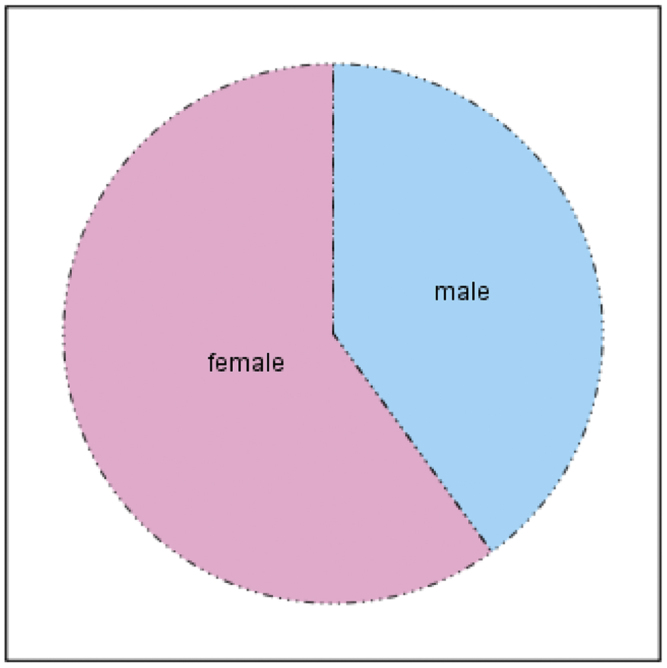
Gender distribution of study sample.

The mean age of the patients was 5.2 months: the majority of patients were within 4 months to 6 months (Fig. 5), 12 patients were male (40%) and 18 were female (60%) (Fig. 6).

Mean alveolar gap (AG) and palatal gap (PG) before the first operation was 10.33 and 12.65 mm, respectively. After the first operation, mean AG become 6.27 mm with a mean reduction of 4.067 mm, while the mean PG was reduced to 8.13 mm with a mean reduction of 4.517 mm (Table 1). In this study, the first operation took a mean time of 65 min, but for the second operation, the mean time was 45 min. Mean total (first and second) operation time was 110 min.

**Table 1:** Alveolar and palatal gap measurements and changes in both of first and second operations.

	Alveolar gap (AG) mm			Palatal gap (PG) mm		
	1st operation	2nd operation	Reduction	1st operation	2nd operation	Reduction
Mean ± SD	10.333 ± 1.6522	6.27 ± 1.437	4.067 ± 0.7958	12.650 ± 1.6461	8.13 ± 1.634	4.517 ± 0.9143
Median	10.750	6.00	4.000	12.750	8.00	5.000

Postoperatively, 3 patients developed a small oronasal fistula that was detected and corrected during the second operation and 1 patient developed partial lip dehiscence. None of the patients had postoperative significant bleeding or infection.

## Discussion

Cleft lip and cleft palate are the most commonly seen congenital malformations of the head and neck. Infants born with a cleft present to the reconstructive surgeon with a uniquely difficult surgical challenge, one that requires both an esthetic sense and technical skill to restore form and function.^11^ Patients who have cleft lip or palate face significant lifelong communicative and esthetic challenges and difficulties with deglutition. Management of patients who have oro-facial cleft requires an understanding of the anatomy and pathophysiology associated with the deformity and developmental difficulties encountered by these patients.^12^ Palatal fistula and transverse growth limitation present a notable problem after palatal surgery regardless of the institution or the type of repair. The main cause of these complications is the lack of tissue, creating tension at the closure, as well as healing by secondary intention and subsequent growth restriction.^13^

Vomerine mucosal flaps can be useful for the closure of particularly wide cleft and bilateral clefts.^14^, ^15^ The primary concern regarding vomer flaps for palatal closure has been their effect on facial growth.^15^ Semb's report on the longitudinal data of the Oslo group is important for those who denounce the vomer flap.^16^ This report demonstrated that the possible growth-retarding effect of a vomer flap has been discussed by several authors who recognized further desirable growth in patients operated without vomer flap; however, this is not a uniform observation in the comparative studies. In the opinion of the Oslo team, a vomer flap provides special benefits of the initial division of the oral and nasal cavities without synthetic obturators, a low rate of symptomatic fistulae, an agreeable arch shape, and a nice base of mix dentition alveolar bone graft.^16^ The advantages of using a vomer flap simultaneously with cleft lip repair include reducing the gap of the palate, which in turn reduces the time and effort required for definitive hard palate closure in the second stage. In addition, it reduces the incidence of postoperative fistula formation.^14^, ^17^, ^18^

In this study, 30 patients with complete cleft lip and palate, their ages ranging between 4 and 8 months, were operated simultaneously by vomer flap. The first stage included cleft lip repair done at the time of presentation, while the second stage involved complete hard and soft palate repair carried out nearly 12–14 weeks after the first surgery.

Both alveolar and palatal gaps were measured preoperatively, the mean alveolar gap (AG1) was 10.33 mm and mean palatal gap (PG1) was 12.65 mm. At the second stage of operation, measurements showed that the mean alveolar gap (AG2) become 6.27 mm with a mean reduction of 4.067 mm, while the mean PG reduced to 8.13 mm with a mean reduction of 4.517 mm. The results of this study were consistent with Noor-Al Ferdous et al.^14^ where 35 patients with complete unilateral cleft lip and palate were subjected to a simultaneous repair of the hard palate by vomer with cleft lip repair as the first stage. Their results showed that the mean alveolar gap was reduced by 5.3 mm and the mean cleft palatal gap was reduced by 4.9 mm after 12–13 weeks from the first surgery. Also, the results of the present study were consistent with Abdelmoktader et al.^19^ who reported that after the first operation, the mean alveolar gap was reduced 4.9 mm and the mean palatal gap was reduced 4.6 mm in their study group of 30 patients with unilateral complete cleft lip and palate repaired simultaneously by vomer flap. In a comparative study by Noor-Al Ferdous KM et al.,^20^ who measured changes in the alveolar gap and palatal gap between two groups of patient, 23 of them had two-stage palatoplasty and 20 patients had single stage palatoplasty, he found that reduction in the alveolar gap was significant in both groups and was more in the two-stage palatoplasty 5.30 mm and was 4.42 mm in single stage palatoplasty group. The Mean reduction in palatal gap in the two-stage group was 4.95 mm while the reduction of palatal gap in the single-stage group was 2.07 mm and none of the patients had a reduction of more than 3.5 mm, they found that palatal gap reduction in the two-stage group was highly significant.

In the second stage of operation, which was done 12–14 weeks after the first stage, the minimal dissection helped us in reducing operation time (average was 45 minutes); also the minimal dissection reduced the bleeding amount. A lateral relaxing incision was used in only 4 cases.

Only 3 cases out of 30 patients developed an oronasal fistula. This complication occurred one patient who had a wide cleft palate gap of 15–16 mm. Another patient developed lip dehiscence following first stage surgery, exactly on the fifth day postoperatively after sustaining direct trauma. Apart from the former mentioned four cases, no major complications were encountered both after the first or the second stages of operation.

Nasal layer closure by vomer flap is simple to execute without adding surgical trauma, and the quality of the tissue is very similar to that of the nasal mucosa. The flap is supple and procurable in the vicinity of the palatal cleft; however, growth pattern has not been assessed in this study, and therefore long-term follow-up should be carried out to assess the anthropometric measurement in order to exclude any deleterious effect after incorporation of the vomerine flap over maxillary growth.

Another point of concern regarding vomerine flap is the risk of ischemia to the bony vomer and premaxilla in cases of bilateral cleft lip and palate.

## Conclusion

This study showed that nasal layer closure by vomer flap to repair hard palate at the time of primary cleft lip repair is effective in reducing both the time and effort of operation in the second stage repair of the cleft palate. The procedure is easy to perform and it reduces both the alveolar and palatal gaps, which facilitates complete cleft palate repair and thereby reduces the chance of oro-nasal fistula formation. We recommend long-term follow-up in order to exclude any harmful effects of vomerine flap dissection that could hinder the growth of the maxilla. Also, we recommend including more patients with bilateral complete cleft lip and cleft palate in future observational studies in order to evaluate the risk of ischemia to the bony vomer and premaxilla.

## Conflicts of interest

The authors declare no conflicts of interest.
